# Interleukin-10 and prostaglandin E_2_ have complementary but distinct suppressive effects on Toll-like receptor-mediated dendritic cell activation in ovarian carcinoma

**DOI:** 10.1371/journal.pone.0175712

**Published:** 2017-04-14

**Authors:** Eva Brencicova, Ann L. Jagger, Hayley G. Evans, Mirella Georgouli, Alex Laios, Steve Attard Montalto, Gautam Mehra, Jo Spencer, Ahmed A. Ahmed, Shanti Raju-Kankipati, Leonie S. Taams, Sandra S. Diebold

**Affiliations:** 1 Peter Gorer Department of Immunobiology, Division of Immunology, Infection and Inflammatory Disease, King’s College London, London, United Kingdom; 2 Centre for Molecular & Cellular Biology of Inflammation (CMCBI), Division of Immunology, Infection and Inflammatory Disease, King’s College London, London, United Kingdom; 3 Nuffield Department of Obstetrics and Gynaecology, University of Oxford, Oxford, United Kingdom; 4 Department of Gynaecological Oncology, St Thomas’ Hospital, London, United Kingdom; Columbia University, UNITED STATES

## Abstract

Dendritic cells (DC) have the potential to instigate a tumour-specific immune response, but their ability to prime naïve lymphocytes depends on their activation status. Thus, for tumour immunotherapy to be effective, the provision of appropriate DC activation stimuli such as Toll-like receptor (TLR) agonists is crucial in order to overcome immunosuppression associated with the tumour microenvironment. To address this, we investigated how ovarian carcinoma (OC)-associated ascites impedes activation of DC by TLR agonists. Our results show that ascites reduces the TLR-mediated up-regulation of CD86 and partially inhibits the production of the pro-inflammatory cytokines interleukin 6 (IL-6), IL-12 and tumour necrosis factor α (TNFα) in monocyte-derived DC from healthy controls. We further observe an impaired T cell stimulatory capacity of DC upon activation with TLR agonists in the presence of ascites, indicating that their functionality is affected by the immunosuppressive factors. We identify IL-10 and prostaglandin E2 (PGE_2_) as the pivotal immunosuppressive components in OC-associated ascites compromising TLR-mediated DC activation. Interestingly, IL-10 is present in both ascites from patients with malignant OC and in peritoneal fluid from patients with benign ovarian conditions and both fluids have similar ability to reduce TLR-mediated DC activation. However, depletion of IL-10 from ascites revealed that the presence of paracrine IL-10 is not crucial for ascites-mediated suppression of DC activation in response to TLR activation. Unlike IL-10, PGE_2_ is absent from peritoneal fluid of patients with benign conditions and selectively reduces TNFα induction in response to TLR-mediated activation in the presence of OC-associated ascites. Our study highlights PGE_2_ as an immunosuppressive component of the malignant OC microenvironment rendering PGE_2_ a potentially important target for immunotherapy in OC.

## Introduction

Chemo-resistance in patients experiencing relapse after conventional therapy is frequent in OC and constitutes an important factor correlating with poor prognosis [1]. Thus there is an urgent need for alternative intervention strategies. Immunotherapeutic induction of anti-tumour immunity represents a promising treatment option in OC [2]. However, in order to develop robust and effective immunotherapy protocols for clinical use, a better understanding of the obstacles for anti-tumour immunity induction in OC posed by the immunosuppressive tumour microenvironment is required.

Serous epithelial OC is the most common histological subtype of OC comprising 85% of all cases. Although an aggressive tumour with invasive potential, its metastases remain largely restricted to the peritoneal cavity even in late clinical stages, frequently accompanied by the formation of ascites. The localization to the peritoneal compartment allows the tumour to create an enclosed immunosuppressive milieu that it can thrive in, and the promotion of such an immunomodulatory local environment is a central mechanism of tumour escape and progression in OC [3–6]. The OC microenvironment represents a complex immunosuppressive network of cytokines and other factors. Many of the immunosuppressive components such as IL-10, transforming growth factor β (TGFβ and leukemia inhibitory factor (LIF) are soluble factors that are abundant in OC-associated ascites [6–9]. Vascular endothelial growth factor α (VEGFα) is part of this immunosuppressive network and promotes tumour growth by inducing angiogenesis and recruiting immature myeloid cells to the tumour tissue [10]. Similarly, pro-inflammatory cytokines such as IL-6 and TNFα that are also present in the OC microenvironment, support tumour progression by influencing angiogenesis and tumour infiltration with myeloid cells [11, 12]. The recruited myeloid cells differentiate into tumour-associated macrophages (TAM) and immature DC characterized by the production of indoleamine 2,3-dioxygenase (IDO), and are capable of inducing regulatory T cells [13].

The peritoneal cavity of OC patients is infiltrated with a variety of leukocyte populations, and the immune cell composition within the tumour microenvironment impacts upon disease progression and has been reported to correlate with clinical outcome [14, 15]. DC are present in the OC environment [16, 17] and as professional antigen-presenting cells (APC) they are thought to be pivotal for the initiation of tumour-specific immune responses because of their ability to take up and process tumour antigens and prime cytotoxic T cell (CTL) responses. However, the ability of DC to launch a potent anti-tumour immune response is dependent on their direct activation via pattern recognition receptors (PRR) such as TLR [18]. Despite the presence of damage-associated molecular patterns with the ability to trigger TLR-mediated responses such as HMGB-1 [19], the tumour microenvironment does not provide agonists for optimal TLR-mediated activation of DC and DC-based therapeutic approaches crucially rely on synthetic TLR agonists as mediators of effective DC activation. In order to devise efficient immunotherapeutic strategies for treatment of OC patients, it is vital to understand how DC integrate the opposing signals provided by strongly stimulating synthetic TLR agonists and immunosuppressive factors associated with the tumour microenvironment.

There are many different DC subsets. Some reside in peripheral tissues and have the ability to migrate to the draining lymph nodes whereas others are resident in lymphoid tissues or circulate in the blood until they are recruited to sites of inflammation [20]. However it is currently still unclear which DC subsets are crucial for the initiation of anti-tumour immunity in response to tumour immunotherapy and different immunotherapeutic strategies may recruit different DC subsets. In this study, we used monocyte-derived DC from healthy donors as a model system to investigate how inflammatory DC integrate concurrent stimulation with TLR-mediated immunogenic and OC-associated immunosuppressive signals.

## Results

### OC-associated ascites partially suppresses TLR-mediated activation of monocyte-derived DC

**Fig 1:**
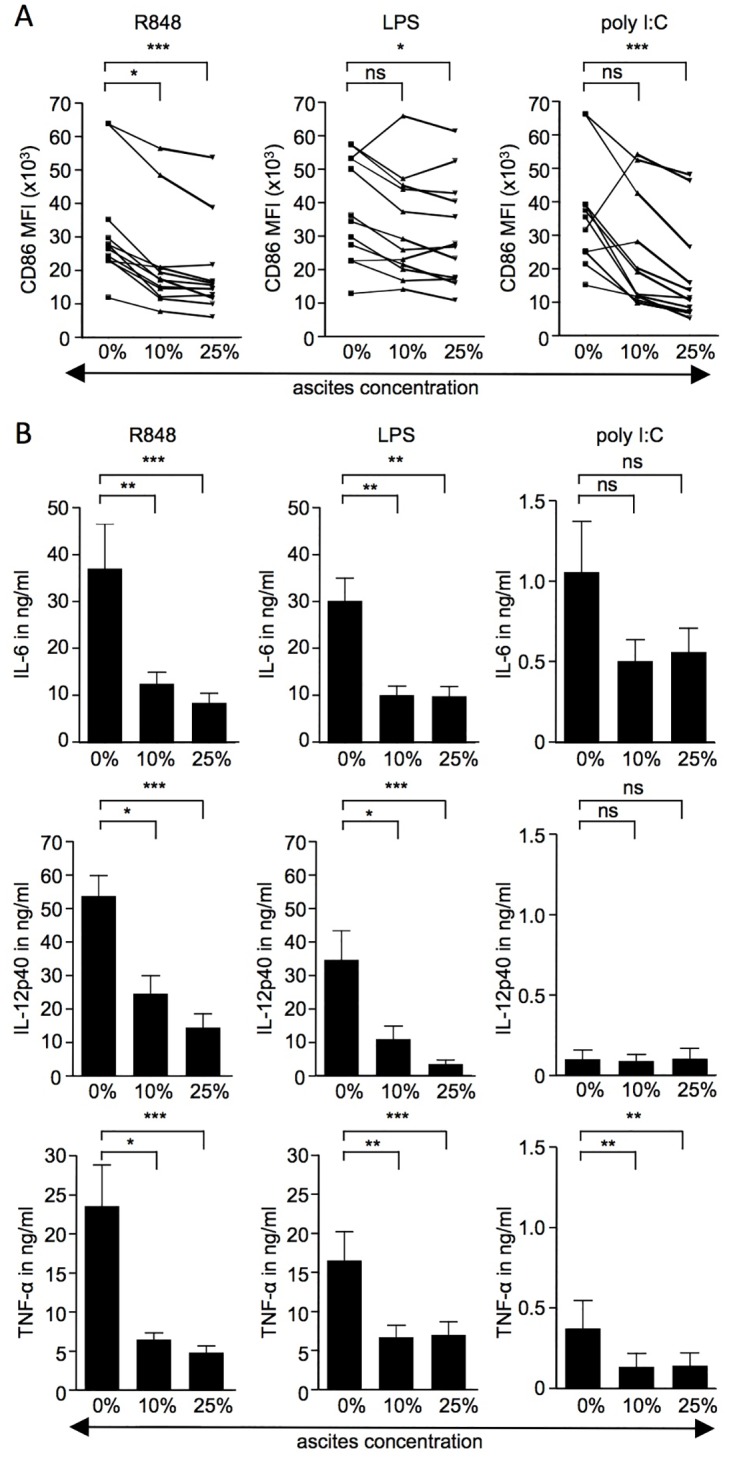
Up-regulations of CD86 and induction of cytokines in response to TLR stimulation in the presence or absence of OC-associated ascites. (A) Monocyte-derived DC were stimulated overnight with 3μg/ml R848, 1μg/ml LPS, 100μg/ml polyI:C in the presence of 0%, 10% or 25% of ascites from patients with malignant OC. The mean fluorescence intensity (MFI) of the surface marker CD86 was assessed by flow cytometry. (B) Monocyte-derived DC were cultured overnight as described above and cytokines were measured in culture supernatants by flow cytomix analysis or sandwich ELISA (IL-12p40). In total, 12 independent experiments were performed (n = 12) with DC from individual healthy volunteers cultured with ascites from 4, 3, 2 or 1 OC patients (n = 1, 1, 1 and 3 healthy volunteers, respectively). One-way ANOVA was used for statistical analysis (Friedman test with Dunn post test): * = p<0.05; ** = p<0.01; *** = p<0.001; ns = not significant.

In order to investigate the immunosuppressive influence of soluble factors associated with the OC microenvironment, we examined the effect of ascites collected from patients suffering from advanced-stage serous epithelial OC on activation of monocyte-derived DC by TLR agonists. The cellular fraction was removed from the ascites samples by centrifugation and only the cell-free ascites was investigated for its immunosuppressive properties. Monocyte-derived DC express TLR3, TLR4 and TLR8 and as such, polyI:C, LPS and R848 were chosen for stimulation of these TLR, respectively. The TLR agonists were used at concentrations optimised for the induction of pro-inflammatory cytokines such as IL-6 and TNFα in overnight cultures. Compared to R848 and LPS, polyI:C induced rather low levels of IL-6 and TNFα and no IL-12p40 (S1 Fig). For all three TLR agonists, the up-regulation of the co-stimulatory molecule CD86 was reduced in the presence of 25% of ascites (Fig 1A). In the presence of 10% ascites, CD86 up-regulation was significantly reduced in response to R848, but not in response to LPS or polyI:C (Fig 1A). This was the case both when monocyte-derived DC from different healthy donors were cultured with ascites from the same OC patient as well as when culturing the same donor’s monocyte-derived DC with ascites from up to four different patients. CD86 levels on monocyte-derived DC cultured without TLR agonists were not affected by the presence of ascites (S2A Fig). In addition to CD86, we monitored changes in the up-regulation of other surface markers such as CD40 and HLA-DR, as well as the immunoregulatory molecules PD-L1 (CD274) and PD-L2 (CD273). While all of these molecules were up-regulated in response to TLR stimulation, their expression at the cell surface was not consistently affected by the presence of ascites (data not shown).

OC-associated ascites showed an inhibitory effect on the production of the cytokines IL-6, IL-12p40 and TNFα in response to R848- and LPS-mediated activation (Fig 1B). For polyI:C-mediated activation a similar trend was observed. However, overall levels of cytokine induction in response to polyI:C was weaker than for R848 and LPS and the reduction in cytokine production in the presence of ascites was only significant for TNFα (Fig 1B). Interestingly, the reduction in cytokine levels in the presence of ascites was not observed for all cytokines that were monitored. IL-1β and IL-10 induced in response to TLR-mediated activation did not show significant reduction in the presence of 25% ascites (S2B Fig and data not shown).

**Fig 2:**
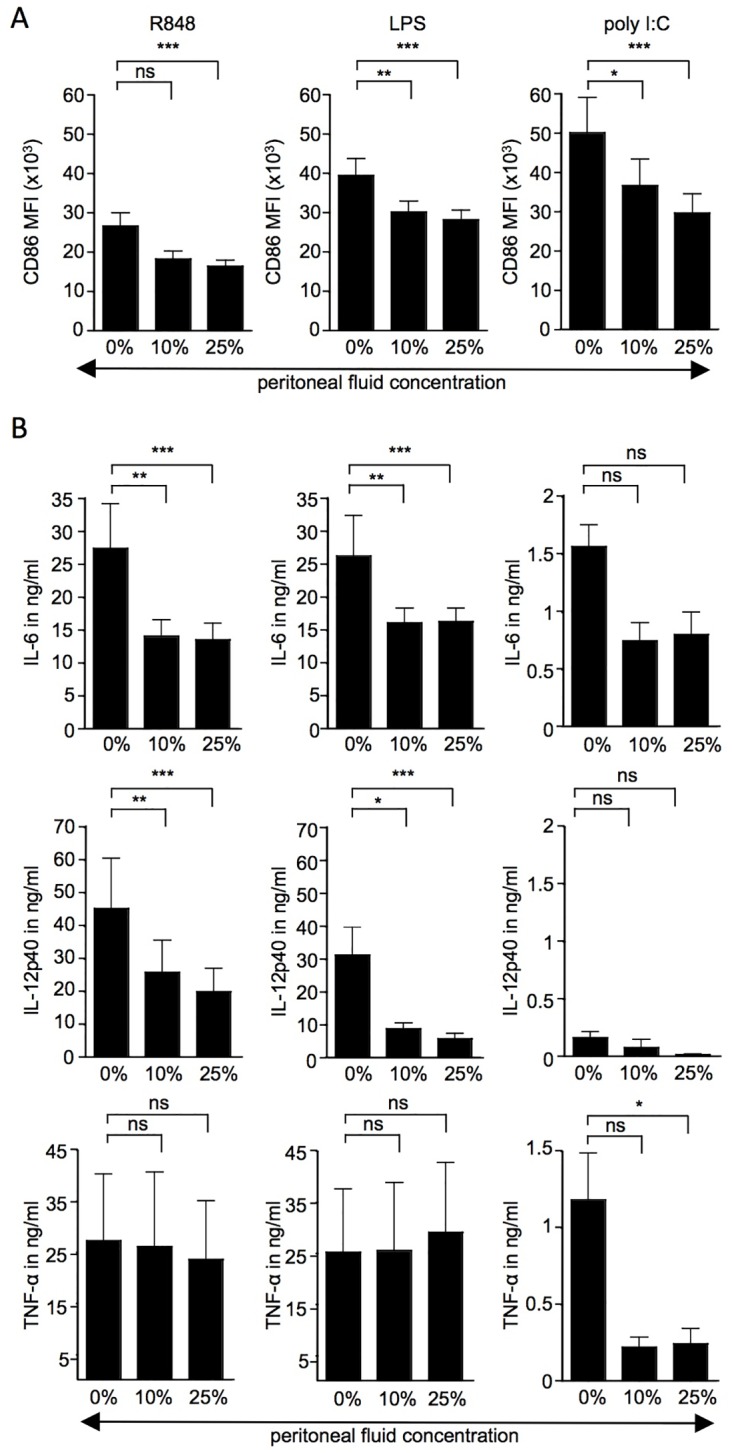
Up-regulations of CD86 and induction of cytokines in response to TLR stimulation in the presence or absence of peritoneal fluid from patients with benign conditions. Monocyte-derived DC were stimulated overnight with 3μg/ml R848, 1μg/ml LPS or 100μg/ml polyI:C in the presence of 0%, 10% or 25% peritoneal fluid obtained from patients suffering from benign ovarian conditions. The following day, (A) the MFI of surface marker CD86 was assessed by flow cytometry and (B) cytokines were measured in culture supernatants by flow cytomix analysis or sandwich ELISA (IL-12p40). In total, 12 experiments (n = 12) were performed with DC from 8 different healthy volunteers cultured with ascites from 2 (n = 4) or 1 (n = 4) patient with benign ovarian conditions. One-way ANOVA was used for statistical analysis (Friedman test with Dunn post test); * = p<0.05; ** = p<0.01; *** = p<0.001; ns = not significant.

We were interested in comparing the immunosuppressive effect of OC-associated ascites to peritoneal fluid from benign ovarian conditions in order to identify markers that distinguish the malignant from the benign microenvironment. Despite the fact that increased levels of peritoneal fluid are usually not observed for benign ovarian conditions, we collected some specimens from patients suffering from benign ovarian conditions, such as fibroadenoma, fibrothecoma, or fimbrial cysts. Like malignant ascites, peritoneal fluid from patients with benign ovarian conditions also reduced up-regulation of the surface marker CD86 and partially inhibited production of the cytokines IL-6 and IL-12p40 (Fig 2). In contrast, TLR agonist-induced TNFα production was largely unaffected by peritoneal fluid from these benign ovarian tumours (Fig 2).

### The immunosuppressive effects correlate positively with the IL-10 levels of OC-associated ascites

**Fig 3:**
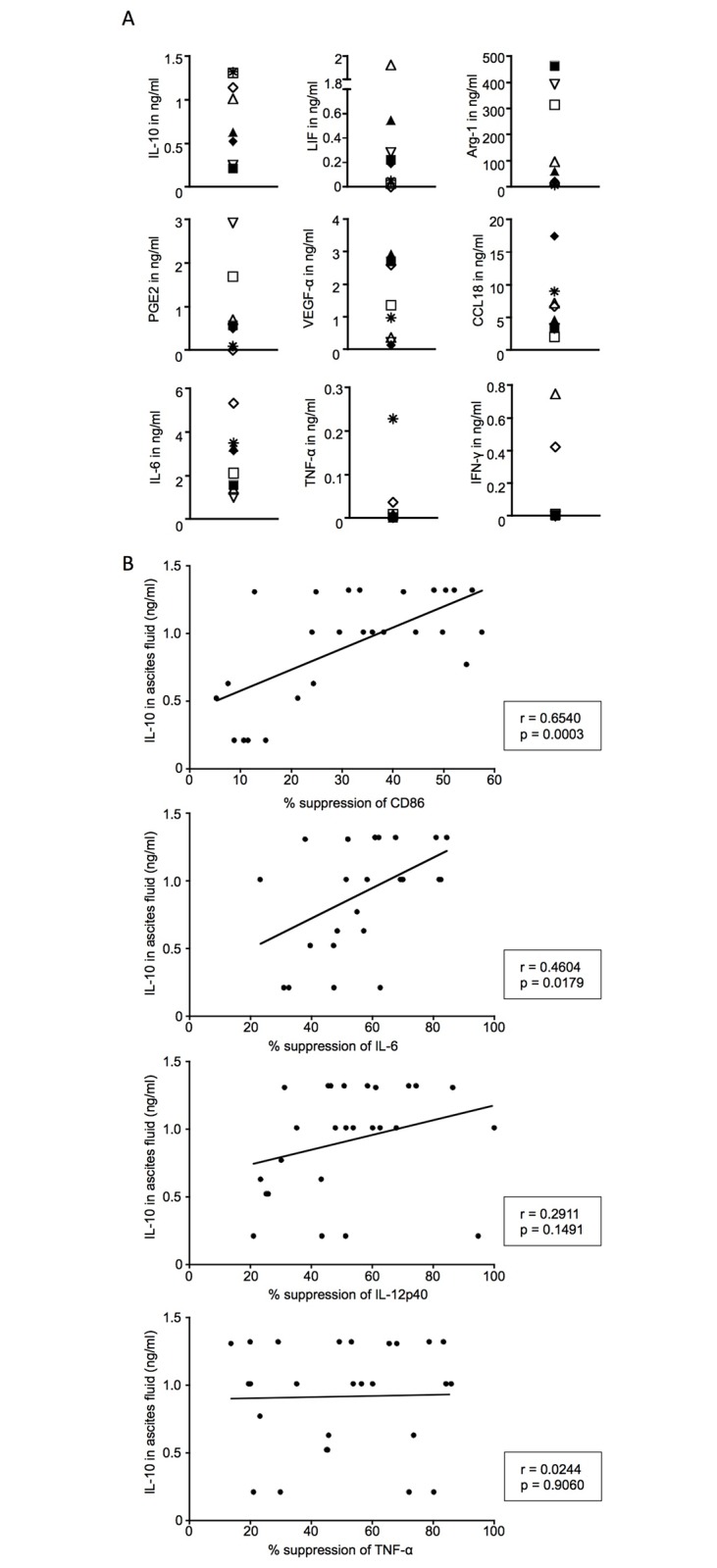
Levels of various immunomodulatory factors in OC-associated ascites and their correlation with the immunosuppressive effects observed in DC activation assays. (A) Protein levels of various factors in ascites samples collected from OC patients were measured by sandwich ELISA. Eight malignant ascites samples used throughout the study are shown. Each symbol represents one patient sample throughout the graphs (n = 8). (B) Levels of IL-10 in ascites samples are correlated to the suppression of TLR-mediated up-regulation of CD86 and production of IL-6, IL-12 and TNFα. Suppression is expressed in per cent reduction of surface marker and cytokine levels when 10% ascites was added to the cell culture as compared to no ascites present; n = 26 (26 independent experiments conducted with monocyte-derived DC from 9 different healthy controls, cultured with ascites from two (n = 4), three (n = 2) or four (n = 3) different OC patients. The Pearson coefficient was calculated to assess statistical significance of correlations.

In order to examine which soluble factors in OC-associated ascites may be responsible for impaired TLR-mediated activation of monocyte-derived DC, we measured the levels of various proteins with known immunosuppressive properties in the collected ascites samples such as IL-10, LIF, Arginase-1 (Arg-1), and PGE_2_. We also determined the concentrations of the angiogenesis-promoting and immunosuppressive factor VEGFα the chemokine CCL18 and the pro-inflammatory cytokines IL-6, TNFα and interferon γ (IFNγ) to further characterise the composition of the OC-associated microenvironment. As expected, the immunosuppressive factors IL-10, LIF, Arg-1 and PGE_2_ were detected in all ascites samples with the protein levels varying considerably between patients (Fig 3A). Similarly, the levels of the pro-inflammatory cytokines IL-6, the chemokine CCL18 and VEGFα differed between samples, creating a unique composition of immunomodulatory factors in each patient (Fig 3A). TNFα and IFNγ levels were low or absent for all ascites samples (Fig 3A). Secreted CTLA-4 was also measured but was not detected in any ascites samples in our study (data not shown).

We investigated whether any of these factors correlated with the observed immunosuppressive effect and detected a strong positive correlation between the levels of the immunosuppressive cytokine IL-10 and the suppressive effect of the ascites samples on CD86 expression and IL-6 production in response to TLR activation (Fig 3B). Similarly, high suppression of IL-12p40 production was consistently observed for ascites samples with high concentration of IL-10, however, due to one value where an ascites sample with comparatively low IL-10 levels suppressed IL-12p40 induction by over 90%, this correlation was not statistically significant (Fig 3B). Interestingly, there was no correlation between TNFα suppression and IL-10 levels in ascites (Fig 3B).

The levels of most other determined proteins found in OC-associated ascites did not correlate with the level of its suppressive properties (S1 Table). However, we observed statistically significant negative correlations between the levels of VEGFα and suppression of CD86 induction, as well as between OC-associated Arg-1 levels and CD86 suppression, and Arg-1 levels and inhibition of IL-6 production (S3 Fig). Although the statistical analysis suggests a positive correlation between TNFα levels in ascites and suppression of CD86, IL-6 and IL-12p40 expression, given that only one sample contained detectable levels of TNFα (Fig 3A), this correlation is likely to be skewed and should, therefore, be disregarded (S3 Fig).

We conclude that levels of IL-10 found in OC-associated ascites positively correlate with the inhibitory properties of ascites on the TLR-mediated up-regulation of the co-stimulatory molecule CD86, as well as the production of the cytokines IL-6 and IL-12p40 but not TNFα.

### The suppressive effect of OC-associated ascites on R848-induced activation of monocyte-derived DC is IL-10 dependent

**Fig 4:**
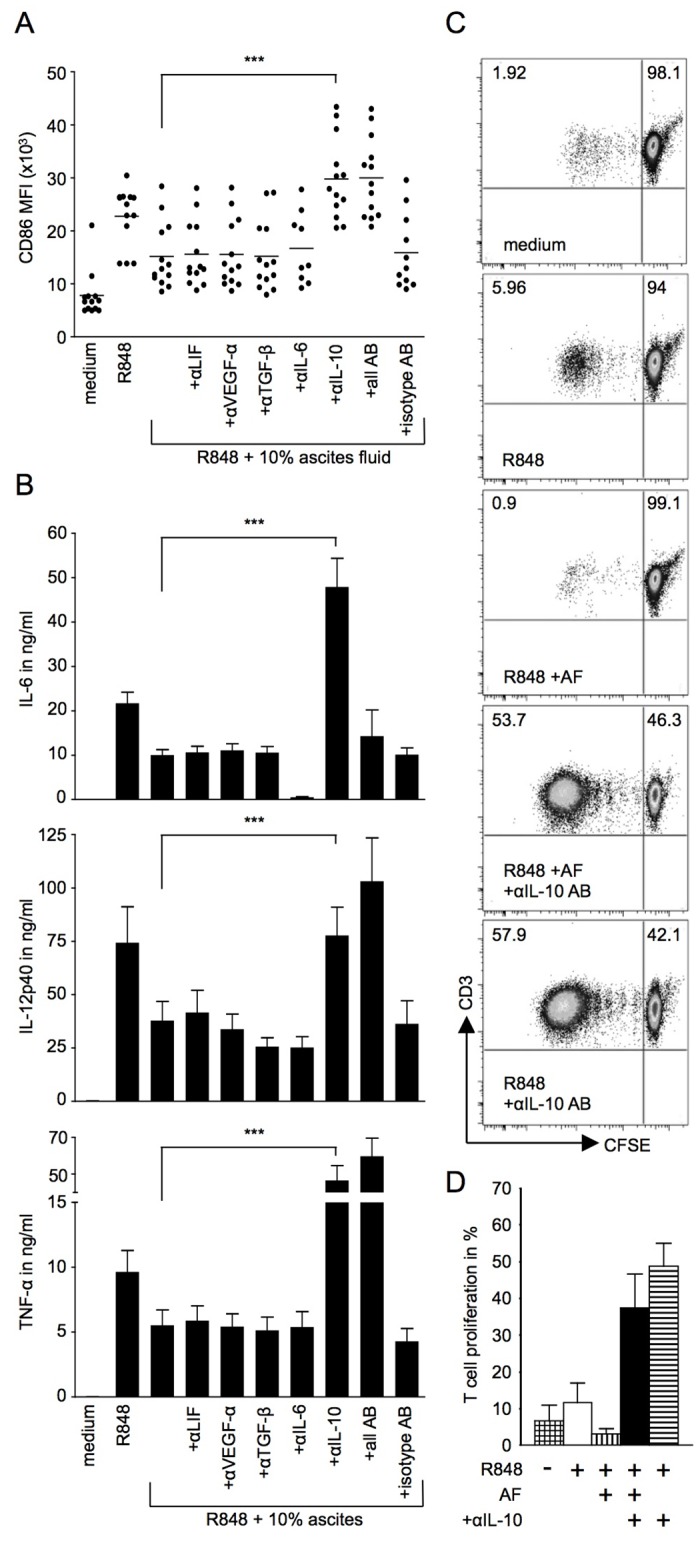
Neutralisation of IL-10 releases the impairment of DC activation in response to TLR stimulation in the presence of OC-associated ascites. (A, B) Monocyte derived DC were cultured in the presence of 3μg/ml R848, 10% ascites and neutralizing antibodies (5μg/ml) as indicated. Neutralizing antibodies were added to cultures alone or in combination. (A) CD86 expression levels were measured by flow-cytometry, and (B) cytokine levels were measured in cell culture supernatants by flow cytomix analysis (IL-6, TNFα) or sandwich ELISA (IL-12p40). n = 9–13 (13 independent experiments; monocyte-derived DC from 8 different healthy donors, cultured with 1 (n = 4), 2 (n = 3) or 3 (n = 1) different ascites samples. IL-6 neutralizing antibody was only used in 9 out of 13 experiments). One-way ANOVA (Friedman test with Dunn post test): *** = p<0.001. Please note that the quantification of IL-6 in samples containing IL-6 neutralizing antibody (αIL-6 and all AB) is compromised. (C, D) For allogeneic MLR, monocyte-derived DC from healthy donors were cultured overnight in complete medium only, or stimulated with 3μg/ml R848 in the presence or absence of 10% ascites fluid (AF) and/or IL-10 neutralizing antibody (5μg/ml) as indicated. Subsequently, such pre-treated DC were washed and co-cultured with CFSE-stained CD4^+^ naïve T cells from another healthy donor at a 1:200 ratio for 6 days. Naïve T cells from the same donor were used consistently for all experiments. The proliferation of T cells was determined by CFSE dilution after 6 days. (C) A representative example of flow-cytometry analysis is shown. (D) Cumulative data from 4 independent experiments showing proliferation in percent of CD3^+^ cells that have undergone at least one round of division. n = 4 (4 independent experiments; monocyte-derived DC from 3 different healthy donors, cultured with ascites from two (n = 1) or one (n = 2) OC patient).

In order to clarify whether IL-10 is mediating the inhibitory effect on TLR-mediated DC activation partially or in full, we added neutralizing antibodies against IL-10 to monocyte-derived DC cultures containing R848 and 10% OC-associated ascites. We chose R848 as TLR agonist for this set of experiments, since it induced the highest levels of cytokine production by DC in the absence of ascites and robust and reproducible suppression of R848-induced cytokine production was observed in the presence of 10% ascites. In parallel, we also tested neutralizing antibodies against IL-6, TGFβ, LIF and VEGFα to identify any contributions to the observed immunosuppressive effect by these factors. IL-6, LIF and VEGFα were present in the ascites samples investigated in this study (Fig 3A). We also included TGFβ, which has been ascribed immunosuppressive roles in different physiological scenarios but the concentration of which we had not determined in the ascites. Upon addition of IL-10 neutralizing antibody, we observed restored CD86 up-regulation and production of IL-6, IL-12p40 and TNFα to or beyond the activation levels of DC stimulated with R848 alone without OC-associated ascites (Fig 4A and 4B) indicating that the observed immunosuppressive effect is indeed IL-10 dependent. None of the other tested neutralizing antibodies had the same effect nor did the combination of neutralizing antibodies show an additive or synergistic effect ruling out a contribution by the other tested immunosuppressive factors in our system (Fig 4A and 4B).

### IL-10 dependent reduction in the T cell stimulatory capacity of monocyte-derived DC in the presence of OC ascites

To investigate whether the IL-10 dependent reduction in monocyte-derived DC activation in the presence of OC-associated ascites had an effect on APC functionality, we performed allogeneic MLR. DC were cultured overnight in medium only or with R848 in the absence or presence of ascites and IL-10 neutralizing antibody, then washed and subsequently co-cultured with allogeneic naïve CD4^+^ T cells for 6 days. T cell proliferation was assessed by CFSE dilution. Our results showed that DC treated with R848 and ascites before the co-culture with T cells exhibited a reduced T cell stimulatory capacity compared to DC pre-treated with R848 alone (Fig 4C and 4D). When IL-10 neutralizing antibody was added to DC cultures alongside ascites, a large increase in T cell proliferation was observed indicating that IL-10-dependent suppression of DC activation impairs their T cell stimulatory activity (Fig 4C and 4D).

To examine whether DC exposure to ascites influenced the type of T cell response induced, intracellular staining of FoxP3 and the cytokines IL-10, IL-17 and IFNγ was performed in these MLR experiments. We saw no alteration in the percentage of FoxP3^+^ or IFNγ^+^ cells among the proliferating T cells after ascites exposure of DC. In all samples only a very small number of T cells produced IL-10 or IL-17 (<1%), with no alteration regardless of ascites exposure of DC (data not shown).

These data show that the inhibitory effects of OC-associated IL-10 on DC activation outlast the period of immediate exposure of DC to this immunosuppressive factor, leading to subsequent impaired T cell stimulatory function of these cells *in vitro*.

### Investigating the effects of autocrine versus paracrine IL-10 on TLR-induced monocyte-derived DC activation in the presence of OC ascites

**Fig 5:**
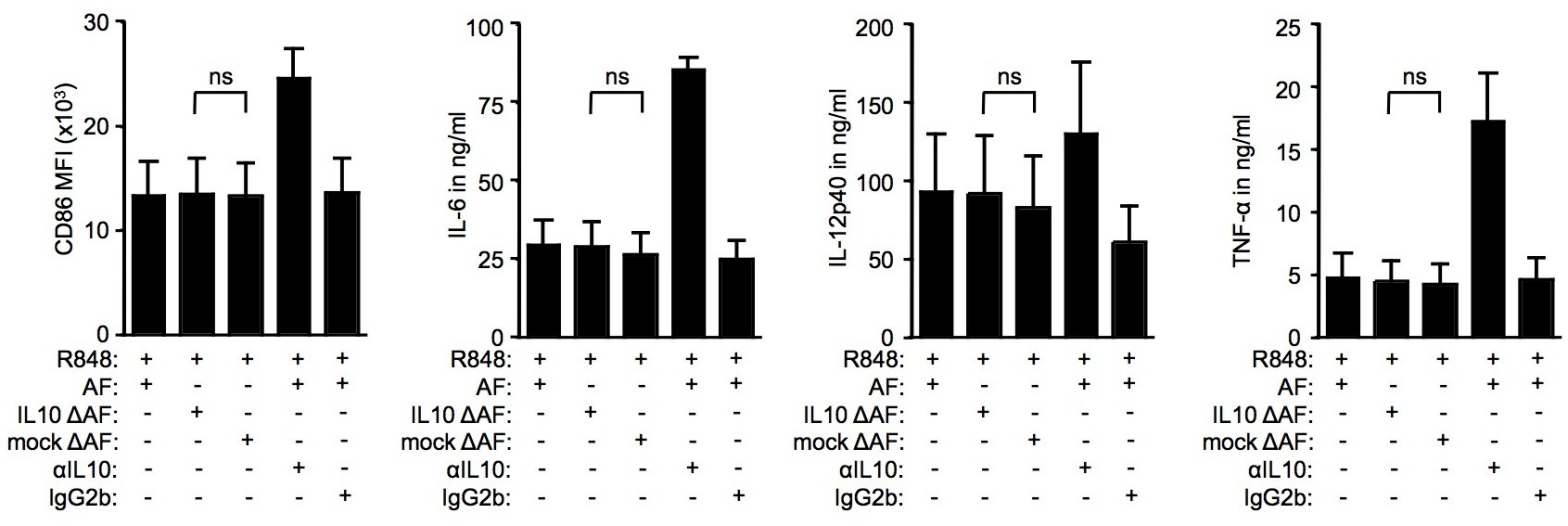
Depletion of IL-10 from OC-associated ascites does not release the suppression of TLR-mediated DC activation. Monocyte-derived DC were stimulated with 3μg/ml R848 in the presence or absence of 10% ascites from ovarian carcinoma patients which had previously been depleted of IL-10 (IL10 ΔAF) or which had undergone mock depletion (mock ΔAF). Alternatively, DC cultures were treated with R848 and untreated ascites (AF) in the presence of αIL-10 neutralizing antibody or isotype control antibody (5μg/ml); n = 3 (3 independent experiments; monocyte-derived DC from 1 healthy donor, cultured with three ascites samples from three individual ovarian carcinoma patients). IL-6 levels were measured in cell culture supernatants by sandwich ELISA.

TLR stimulation induces the production of pro-inflammatory cytokines but also IL-10 in DC [21]. The negative feedback of autocrine IL-10 limits the release of DC-derived pro-inflammatory cytokines at high TLR agonist concentrations and is thought to protect the host from the adverse effects of excessive immune activation [22]. We wanted to confirm the role of OC-associated IL-10 by distinguishing the influence from paracrine versus TLR-induced autocrine IL-10 in our experimental system. To this end we depleted ascites of IL-10 and performed DC activation experiments [23]. To our surprise, DC activated in the presence of IL10-depleted ascites did not show a release of suppression with regard to CD86 up-regulation and cytokine induction in comparison to untreated or mock-treated ascites induction (Fig 5). The result suggests that paracrine OC-associated IL-10 does not impair TLR-mediated DC activation and that the increase in DC activation in the presence of IL-10 neutralizing antibody is largely dependent on inhibition of autocrine IL-10. It also implies that immunosuppressive factors other than IL-10 are responsible for the impairment of DC activation in the presence of ascites.

**Fig 6:**
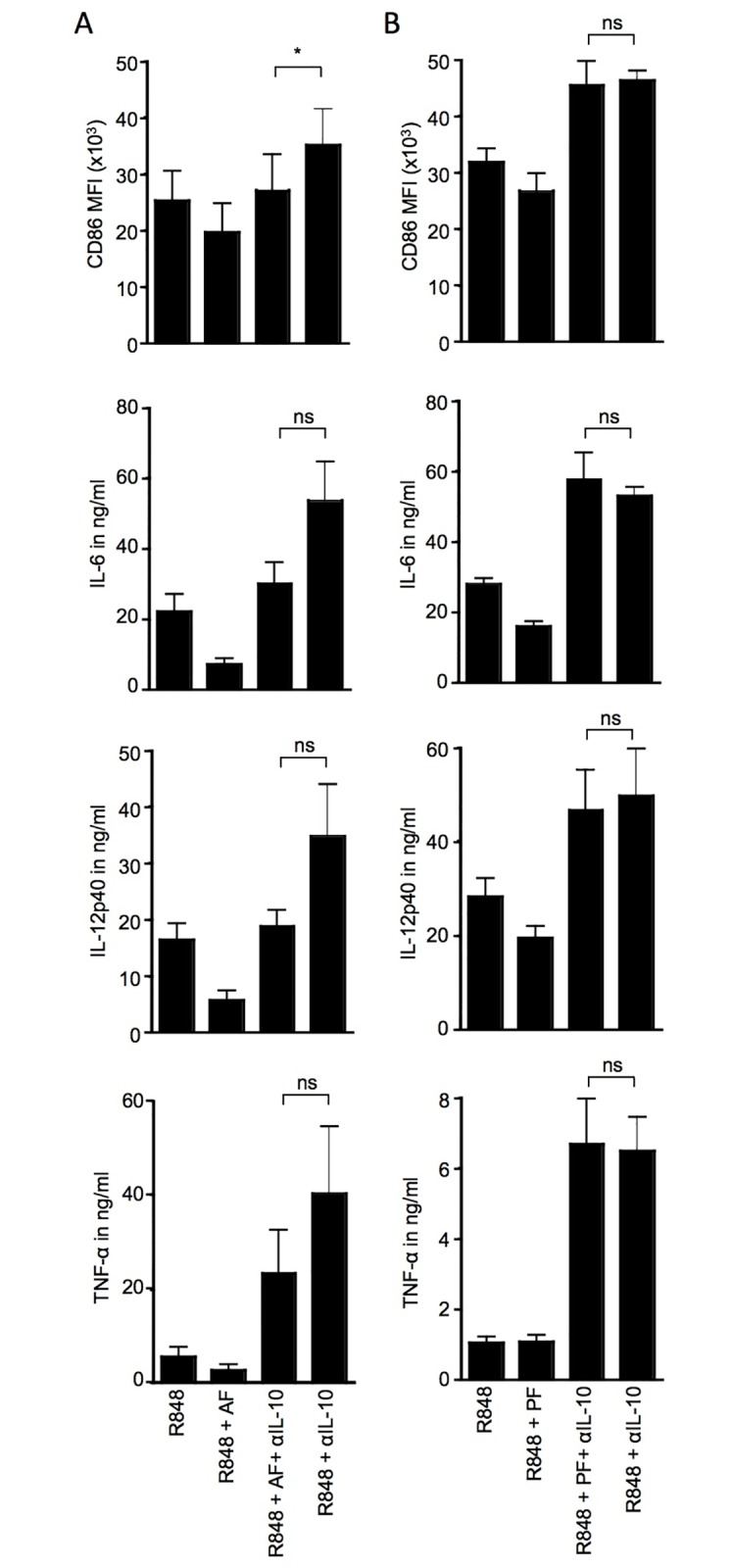
The suppression of DC activation by peritoneal fluid from patients with beningn conditions is fully mediated by IL-10 unlike the suppressive activity of OC-associated ascites. Monocyte-derived DC were stimulated overnight with 3μg/ml R848 in the presence or absence of (A) 10% malignant ascites from OC patients or (B) 10% peritoneal fluid from benign ovarian tumours and IL-10 neutralizing antibody (5μg/ml) as indicated. CD86 expression levels were assessed by flow cytometry, and cytokine levels were measured in cell culture supernatants by flow cytomix analysis (A: IL-6 and TNFα) or sandwich ELISA (A: IL-12p40 and B: TNFα, IL-6 and IL-12p40); for malignant samples n = 6 (6 independent experiments; monocyte-derived DC from 5 different healthy donors, cultured with 1 (n = 4), or 2 (n = 1) different ascites samples); for benign samples n = 9 (9 independent experiments; monocyte-derived DC from 5 different healthy donors, cultured with 1 (n = 3), or 3 (n = 2) different peritoneal fluids); One-way ANOVA (Friedman test with Dunn post test): * = p<0.05, ns = not significant.

In order to confirm the presence of one or more additional immunosuppressive factors, we neutralized IL-10 in DC cultures activated with R848 in the presence and absence of ascites and compared the levels of CD86 up-regulation and cytokine induction between these two treatment conditions. Upon neutralization of IL-10 in DC cultures stimulated with R848 in the absence of ascites (R848 +αIL-10) the expression of the monitored DC activation markers (CD86, IL-6, IL-12p40 and TNFα) was consistently higher than observed for DC stimulated with R848 in the presence of ascites and IL-10 neutralizing antibody (R848 +AF +αIL-10) even though some of these results were not statistically significant (Fig 6A). This observation supports the notion that at least one other immunosuppressive factor impairing DC activation in addition to IL-10 is present in OC-associated ascites. Interestingly, we could not detect a similar difference in DC activation in cultures activated with R848 and IL-10 neutralizing antibodies in the presence and absence of peritoneal fluid from benign ovarian conditions (Fig 6B). This suggests that in contrast to malignant OC, IL-10 acts as a sole agent in impairing TLR-mediated DC activation in the presence of peritoneal fluid from benign ovarian conditions. The levels of IL-10 in peritoneal fluid from benign ovarian conditions varied between patients. While the average amount of IL-10 in ascites was higher than in peritoneal fluid, this difference was not significant (S4 Fig). However, this could be a result of the limited number of peritoneal fluids that were investigated for IL-10 levels.

### PGE_2_ and IL-10 in OC-associated ascites have an additive effect on suppression of TLR-mediated TNFα induction

**Fig 7:**
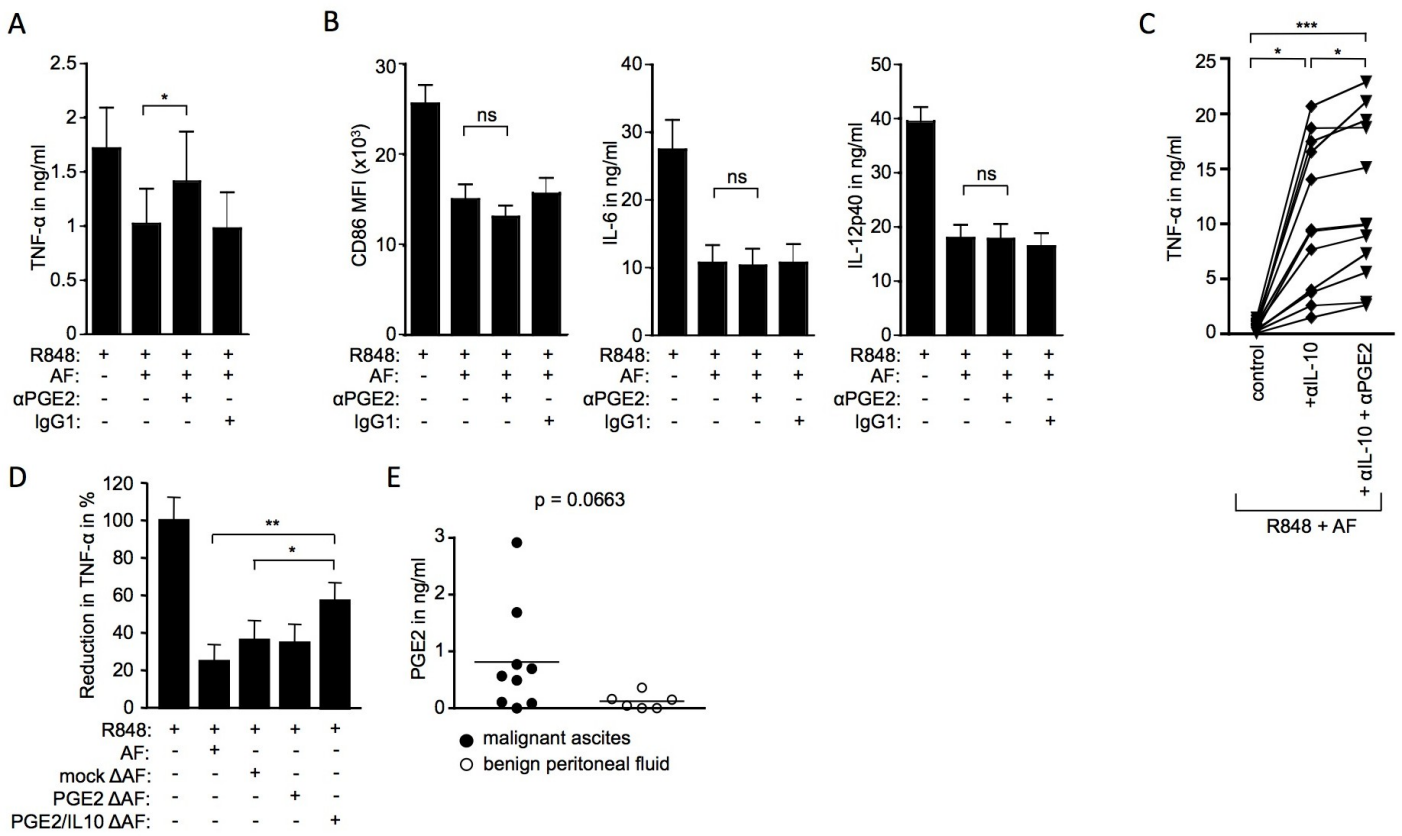
Paracrine OC-associated PGE_2_ impairs TLR-mediated DC activation and distinguishes malignant carcinoma from benign ovarian conditions. Monocyte-derived DC were stimulated overnight with 3μg/ml R848. (A-B) Malignant ascites and neutralizing antibodies (5μg/ml) against PGE_2_ were added as indicated; n = 11 (11 independent experiments; monocyte-derived DC from 4 different healthy donors, cultured with 2 (n = 1), or 3 (n = 3) different ascites samples) (C) Neutralizing antibodies against IL-10 and PGE_2_ were added as indicated to cultures containing 3μg/ml R848 and 10% ascites; n = 9 (9 independent experiments, monocyte-derived DC from 4 different healthy controls, cultured with 1 (n = 1), 2 (n = 1) or 3 (n = 2) different ascites samples). (D) Malignant ascites which had previously been depleted of PGE_2_ (PGE2 ΔAF) or PGE_2_ and IL-10 (PGE2/IL10 ΔAF) or which had undergone mock depletion (mock ΔAF) was added as indicated. As control, DC cultures were treated with R848 and untreated ascites (AF); n = 4 (4 independent experiments; monocyte-derived DC from 1 healthy donor, cultured with ascites samples from two individual ovarian carcinoma patients). The level of TNFα is expressed in relation to the TNFα levels induced in response to LPS, which were set to 100%. (A-D) Cytokine levels in cell culture supernatants were measured by sandwich ELISA. One-way ANOVA (Friedman test with Dunn post test); * = p<0.05; ** = p<0.01; *** = p<0.001; ns = not significant. (E) PGE_2_ levels in malignant ascites (n = 9) and benign peritoneal fluid (n = 6) were measured by sandwich ELISA. Mann-Whitney U test was used for statistical analysis.

To elucidate which factors present in ascites distinguish the malignant OC environment from the environment of benign ovarian tumours, we investigated which proteins in addition to IL-10 in ascites exerted a suppressive influence on TLR-mediated DC activation. Given the absence of TNFα suppression by benign peritoneal fluid in response to R848 and LPS (Fig 2B), and the lack of correlation between TNFα suppression and IL-10 levels in ascites (Fig 3B), we focussed on proteins that have previously been shown to regulate TNFα production. PGE_2_ represented a promising candidate, because monocyte-derived DC express the PGE_2_ receptors EP2 and EP4, and PGE_2_ has been described to regulate cytokine production in DC including TNFα [24, 25]. To investigate whether PGE_2_ present in ascites inhibits TNFα induction, we neutralized PGE_2_ in cultures of cells stimulated with R848 in the presence of OC-associated ascites. Indeed, neutralization of PGE_2_ significantly alleviated the ascites-induced suppression of TNFα induction upon TLR stimulation (Fig 7A). This effect was specific to TNFα amongst the activation markers examined, with the co-stimulatory molecule CD86 and the cytokines IL-6 and IL-12p40 remaining unaffected by PGE_2_ neutralizing antibody (Fig 7B). To exclude the possibility that the observed effect was caused by neutralization of autocrine PGE_2_ produced by DC upon TLR activation, we neutralized PGE_2_ in R848-activated cultures without ascites. We saw no effect of PGE_2_ neutralization on TNFα levels in response to R848-mediated activation (S5 Fig), which suggests that autocrine PGE_2_ does not play a role in the suppression of TLR-induced TNFα production by monocyte-derived DC.

Since we had previously also observed an alleviation of TNFα suppression by IL-10 neutralizing antibodies (Fig 4B), we were interested to see whether IL-10 and PGE_2_ in OC-associated ascites acted synergistically or additively. Our data show that while IL-10 is the major inhibitor of TNFα induction, PGE_2_ has an additive effect on TNFα suppression (Fig 7C). In order to evaluate the role of OC-associated PGE_2_, we depleted ascites samples of PGE_2_ alone or of PGE_2_ and IL-10 together and examined TNFα release in the presence and absence of these OC-associated suppressive factors. DC stimulated with R848 in the presence PGE_2_-depleted ascites showed levels of TNFα production equivalent to mock-depleted samples (Fig 7D). However, the suppressive activity of ascites depleted of PGE_2_ and IL-10 was significantly reduced compared to mock-treated or untreated samples while PGE_2_ depletion alone had no such effect (Fig 7D). This finding not only underlines a particular immunosuppressive role of PGE_2_ in OC, it also rehabilitates a suppressive role of OC-associated IL-10. It is currently unclear why IL-10 depletion in the absence of PGE_2_ depletion fails to reveal the immunosuppressive activity of OC-associated IL-10 in our experimental system.

To further confirm PGE_2_ as a factor that distinguishes the malignant OC environment from that of benign ovarian tumours, we measured the levels of PGE_2_ in malignant ascites and benign peritoneal fluid samples used in our study (Fig 7E). The levels of PGE_2_ in malignant ascites vary between patients and while some samples were negative for this protein, most malignant ascites samples contained detectable levels of PGE_2_. Interestingly however, all samples of peritoneal fluid from benign ovarian conditions showed low or non-detectable levels of PGE_2_. Hence, our data suggest PGE_2_ is a factor absent from benign ovarian conditions that can be present in the malignant OC environment and that specifically impairs TLR-mediated TNFα induction by DC.

## Discussion

In this study, we have examined the immunosuppressive influence of soluble factors in ascites from OC patients on TLR-mediated activation of monocyte-derived DC *in vitro*. We have observed partially impaired up-regulation of the co-stimulatory molecule CD86 and reduced production of the cytokines IL-6, IL-12p40 and TNFα when DC are activated with TLR agonists in the presence of ascites. Co-stimulatory molecules and pro-inflammatory cytokines are generally used as surrogate markers for the T cell priming activity of DC and their up-regulation typically correlates well with the T cell stimulatory activity of these antigen-presenting cells. Interestingly, the suppression was specific to these activation markers, with other monitored markers such as HLA-DR, CD40, IL-1β as well as immunoregulatory molecules such as PD-L1 and PD-L2 and IL-10 showing a high variability between donors and no consistent patterns of suppression or up-regulation. DC activated in the presence of ascites displayed reduced T cell stimulatory activity, showing that the selective suppression of activation markers in the presence of ascites was sufficient to partially compromise the functionality of DC as antigen presenting cells *in vitro*.

Identification of the components in the OC environment that contribute to the observed suppressive effect is an important step towards optimization of DC-based immunotherapeutic protocols that employ TLR agonists as adjuvants in support of T cell priming. Monocyte-derived DC are believed to represent inflammatory DC phenotypically and functionally and are distinct from primary DC subsets. It is currently unclear how TLR-mediated activation of primary DC subsets is affected by immunosuppressive factors in the OC microenvironment. The different primary DC subsets have diverging TLR expression profiles with plasmacytoid DC being for example particularly specialised in sensing viral infections [26]. However, independent of their TLR expression profile and their responsiveness to particular TLR agonists, the mechanisms that impair TLR-mediated DC activation in the tumour microenvironment will depend on the expression of the receptors for individual OC-associated immunosuppressive factors and their downstream signalling cascades. These will have to be investigated individually for the different DC subsets to complete the whole picture. Noteworthy, inflammatory DC are thought to be particular strong inducers of T cell activation. Safeguarding and optimising their activation during immunotherapeutic intervention is likely to have beneficial outcomes for therapeutic strategies aiming to capitalize on their antigen-presenting and T cell-priming functionality. By selectively blocking a range of immunosuppressive factors with neutralizing antibodies, we have shown that OC-associated IL-10 and PGE_2_ are pivotal to suppressing TLR-mediated DC activation *in vitro*.

The immunosuppressive role of IL-10 in OC is well documented, with OC patients showing elevated IL-10 levels in sera in comparison with healthy controls and women with benign ovarian tumours [7]. Importantly, IL-10 levels in both serum and ascites correlate with advanced disease and drop significantly after de-bulking surgery [7, 27]. Several cell types within the OC environment secrete IL-10, with TAM and regulatory T cells being major contributors [6, 28]. Equally, epithelial OC cells show higher IL-10 production compared to normal ovarian epithelium [29]. Unexpectedly, in our experimental system we could not confirm that paracrine OC-associated IL-10 rather than autocrine IL-10 affects TLR-mediated DC activation. This is somewhat counterintuitive since paracrine IL-10 has been shown to have a dose-dependent suppressive effect on DC and it is difficult to reconcile with findings that OC-associated IL-10 correlates with clinical prognosis [7, 30]. The mechanistic basis for this result and its correct interpretation are currently unclear. Interestingly, when depleting ascites samples of IL-10 and PGE_2_ simultaneously, we observed a significant reduction in ascites-induced suppression of DC activation suggesting that OC-associated IL-10 may act synergistically with PGE_2_. A possible explanation could be that when depleting paracrine IL-10, paracrine PGE_2_ may still be able to act suppressive in combination with autocrine IL-10 even though autocrine IL-10 becomes available only later upon TLR-mediated activation. The effect of depleting paracrine PGE_2_ alone may be too weak to be noticed, masked by the suppressive activity of paracrine and autocrine IL-10. However, when paracrine IL-10 and PGE_2_ are depleted together releasing early suppression of activation of DC before TLR-induced IL-10 production kicks in, their synergistic effects could become obvious.

Interestingly, IL-10 levels in OC-associated ascites did not significantly differ from levels in peritoneal fluid from patients with benign ovarian conditions. Furthermore, we could demonstrate that IL-10 neutralization releases most or all of the suppression of TLR-mediated DC activation by ascites and peritoneal fluid, respectively. This would suggest that IL10-mediated immunosuppression is similarly present in benign and malignant tumour microenvironments and while clearly contributing to impaired immune responsiveness is not a defining driving force behind tumor progression in the malignant tumour environment. In contrast, PGE_2_ which was missing from the peritoneal fluid samples that we have investigated and may represent an immunosuppressive driver that defines a malignant tumour microenvironment.

Even though IL-10 may not be driver of malignancy, in light of our and other laboratories’ data, reducing IL-10 in the OC environment could potentially enhance immunotherapeutic strategies in OC patients, especially those aiming to activate DC *in situ* by *in vivo* targeting. The reduction of IL-10 in serum and ascites as observed after de-bulking surgery offers an interesting window of opportunity, which should be considered when designing the timeframe of DC vaccination studies. Systemic IL-10 blockade bears a significant risk of adverse autoimmune reactions, which makes any such approach problematic, although interestingly, a small study administering neutralizing IL-10-specific antibodies to six systemic lupus erythematosus patients showed that this was surprisingly well tolerated [31]. Nevertheless, the associated risks should not be underestimated and clinical trials using this protocol in larger cohorts may not be feasible. However, since OC metastases are generally restricted to the peritoneal cavity even in late clinical stages, an i.p. administration of IL-10-specific antibodies or IL-10R blockers may hold promise and may be an approach worth exploring in *in vivo* models of murine OC where IL-10 signalling has also been shown to be of central importance to the regulation of immune responses within the tumour environment [32].

While IL-10 levels in OC-associated ascites correlated with a reduced induction of most DC activation markers in our study, IL-10 levels did not correlate with the suppression of TNFα production indicating the presence of at least one other immunosuppressive factor. Further blocking experiments with neutralizing antibody revealed that OC-associated PGE_2_ selectively reduces TLR-induced TNFα induction in DC, but does not affect any of the other activation markers. PGE_2_ is an inflammatory mediator with known immunosuppressive function and has been shown to be crucial for DC migration and to influence cytokine release in response to TLR activation [24, 25, 33]. Tumour-derived PGE_2_ has been shown to synergize with TGFβ in inhibiting interferon α production by plasmacytoid DC [34]. The levels of TGFβ present in the OC-associated ascites samples tested in this study are unknown. However, TGFβ is unlikely to play a role in our study, since neutralizing TGFβ-specific antibodies had no effect on DC activation in the presence of ascites and since the main immunosuppressive factor compromising TLR-mediated DC activation in our study was IL-10. In other studies, it was shown that PGE_2_ is crucial for induction of IDO in DC and, thus, promotes the induction of regulatory T cells [35, 36]. In our study we were unable to detect IDO induction by DC in the presence of ascites and we did not observe the induction of FoxP3^+^ T cells in the allogenic MLR assay (data not shown). The discrepancies in the observed PGE_2_-mediated effects on DC activation between different studies are very likely a consequence of differences in the concentration of PGE_2_ and in the presence or absence of other factors influencing DC activation. The low levels of PGE_2_ present in our DC cultures (≤0.75ng/ml) in combination with the presence of high levels of IL-10 and strong TLR agonists seem to create an environment in which PGE_2_ affects TNFα induction but has no significant effect on other activation markers such as IL-12p40 and IL-6 or the induction of IDO. The selectivity of PGE_2_-dependent supression of DC activation is remarkable. There is evidence from a study of monocytes from trauma patients that points towards the regulation of TNFα induction by PGE_2_, though the precise mechanism seems to be very complex and not entirely understood [37]. Similarly, it was shown in rodents that PGE_2_ can suppress the release of TNFα from macrophages after TLR-stimulation [38, 39].

In order to clarify whether the two identified immunosuppressive pathways are unique for the malignant OC environment, we investigated whether IL-10- and/or PGE_2_-mediated inhibition of DC activation is observed when using peritoneal fluid from patients with benign ovarian conditions. Interestingly, impaired TLR-mediated up-regulation of CD86 and the cytokines IL-6 and IL-12p40 was observed in the presence of peritoneal fluid associated with benign ovarian conditions, however, TNFα induction was largely unaffected. When dissecting the factors that mediate the observed effect, it became evident that immunosuppression by peritoneal fluid from benign conditions is largely dependent on IL-10 and independent of PGE_2_. This observation was further supported by the fact that peritoneal fluid from patients with benign ovarian conditions contains levels of IL-10 similar to that observed for OC-associated ascites, but is largely devoid of PGE_2_. Thus, these data suggest that PGE_2_ may be a unique marker of malignant ovarian conditions.

Reports supporting the notion that PGE_2_ is associated with the malignant tumour microenvironment have emerged in the recent past [40]. PGE_2_ has been found to contribute to the immunosuppressive microenvironment by inducing myeloid-derived suppressor cells (MDSC) and suppressive M2 macrophages in the tumour tissue [41, 42]. PGE_2_ levels in OC-associated ascites are elevated due to overexpression of the key enzymes of PGE_2_ synthesis cyclooxygenase-1 and -2 (COX-1 and COX-2, respectively) in cancerous tissue of malignant ovarian tumours as compared to normal ovarian tissue and benign tumours [43]. Inhibitors of COX-1 and COX-2 are a widely used class of drugs referred to as non-steroidal anti-inflammatory drugs (NSAID) and are regarded as potentially powerful drugs for treatment of malignant conditions [44]. NSAID potently block the synthesis of eicosanoids including PGE_2_ and are applied in a variety of medical conditions such as rheumatic diseases. The use of COX inhibitors in cancer therapy and prevention is currently being explored [45] and interestingly, COX inhibitors have previously been used successfully in murine experimental models of breast and lung tumours to enhance DC-based vaccines [46, 47]. In light of our findings, the application of COX inhibitors may equally be an attractive measure to boost the efficacy of DC vaccines in OC.

In this context it is also important to point out that PGE_2_ was not detected in ascites samples from all OC patients indicating that there may be two different patient groups for which PGE_2_-mediated immune suppression either does or does not play a role in driving tumour progression. Also, most likely due to this segregation of OC patients into groups with PGE_2_-containing versus PGE_2_-devoid ascites, we could not detect a correlation between the levels of PGE_2_ in ascites and the suppression of TNFα upon TLR-mediated DC stimulation and a larger sample cohort will be required to revisit this point. It would be interesting to identify whether the presence versus absence of PGE_2_ in the tumour microenvironment influences patient prognosis in general, patient susceptibility to treatment with COX inhibitors and/or responsiveness to certain immunotherapeutic intervention strategies. Much larger patient cohorts will be necessary to address these important questions.

Finally, another question to address in future studies is how our findings in monocyte-derived DC translate into experimental settings of TLR-mediated activation of primary DC subsets and in particular peritoneal DC under the influence of the immunosuppressive OC environment. All our experiments have been performed with DC that were exposed to ascites and TLR agonists simultaneously. It will be important to confirm that DC pre-exposed to the immunosuppressive tumour microenvironment respond in the same or a similar manner to TLR stimulation. However, it is worth taking into account that the generation of inflammatory DC may be induced upon application of TLR agonists in response to immunotherapeutic intervention and thus these DC may not be exposed to the immunosuppressive tumour microenvironment in the same way as primary DC. Exploring these questions will allow for a better understanding of the potential to activate DC *in situ* and bears great significance for the optimization and enhancement of DC-based therapies in OC. While optimising the activation of DC is essential for DC-based therapies, it will be equally important to take into account the activation of other accessory cell types in OC. The immunosuppressive activity of not only IL-10 and PGE_2_ but also other OC-associated factors on such accessory cell types will negatively influence the induction and the effectiveness of anti-tumour immunity. Ultimately, the optimal activation of DC and other hematopoietic and non-hematopoietic cell types will be important for the development of effective anti-tumour immunity.

## Material and methods

### Patient specimens and Peripheral Blood Mononuclear Cells (PBMC) from healthy donors

Ascites from patients suffering from advanced stage (FIGO stage III and IV) serous epithelial ovarian carcinoma was collected by paracentesis (n = 9: St Thomas’ Hospital, London, UK). No information was available regarding the treatment that the patients had received prior to paracentesis. Peritoneal fluid from patients with benign ovarian conditions (fibroadenoma n = 1, fibrothecoma n = 2, benign cyst n = 2 or cystadenoma n = 1) was collected during surgery (St. Thomas Hospital, London and Nuffield Department of Obstetrics and Gynecology, Oxford, UK). Upon receipt, samples were centrifuged and the non-cellular supernatant was aliquoted and stored at -80°C. Upon thawing before use in cell cultures, fluid was passed through a sterile filter with 20μm pore size (Corning Inc., Amsterdam, The Netherlands).

Peripheral blood from healthy volunteers was obtained by venipuncture and collected in heparinized vacutainer tubes (BD, Oxford, UK). PBMC were isolated by density gradient centrifugation using Lymphocyte Separation Medium 1077 (PAA Laboratories, Yeovil Somerset, UK). After centrifugation, the PBMC fraction was washed twice with PBS and live cells were counted in a Neubauer counting chamber.

The collection of ascites, peritoneal fluid and blood samples for this study was approved by the Guy’s Research Ethics Committee (REC Number: 09/H0804/45) and the Berkshire Research Ethics Committee (REC Number: 11/SC/0014 as part of the Oxford Gynaecological Oncology Target Validation Study GO-Target-01). The Guy’s Research Ethics Committee approved this study in its entirety including the consent procedure (REC Number: 09/H0804/45). Informed consent was obtained from all participants in written form and archived locally.

### Generation of monocyte-derived DC

CD14^+^ monocytes were isolated from PBMCs by negative selection using the EasySep^®^ Human Monocyte Enrichment Kit (StemCell Technologies, Grenoble, France) following the manufacturer’s instructions. The purity of the enriched CD14^+^ monocytes was assessed by flow cytometry and was typically over 95%. Enriched CD14^+^ monocytes were seeded at a density of 6-9x10^6^ cells/well in 3ml complete medium (RPMI 1640 (Gibco Life Technologies) containing 10% FCS (Sigma Aldrich), 100U/ml penicillin/streptomycin, 2mM Glutamine, 50μM β mercaptoethanol (all Invitrogen, Paisley, UK). Granulocyte-macrophage colony stimulating factor (GM-CSF) and interleukin-4 were both added at a concentration of 20μg/ml (both R&D Systems, Abingdon, UK) and cells were cultured at 37°C in a 5% CO_2_ atmosphere for 7 days. GM-CSF and IL-4 were replenished on day 2 and day 5 along with 500μl fresh medium. Cells were harvested on day 7 of culture. The differentiation of monocytes into monocyte-derived DC was verified by flow cytometry analysis of the surface markers CD14 and CD1a.

### Activation assay

Monocyte-derived DC were seeded at a density of 1x10^5^ cells/well in 200μl complete RPMI 1640 medium in 96-well plates. All samples were performed in triplicates. TLR agonists were added at optimized concentrations of 3μg/ml for imidazoquinoline (R848; Invivogen, Toulouse, France), 1μg/ml for lipopolysaccharide (LPS; Invivogen) and 100μg/ml for polyinosinic:polycytidylic acid (polyI:C; GE Healthcare, Amersham, UK). Where indicated, 10% or 25% of sterile filtered, non-cellular ascites from patients with malignant OC or peritoneal fluid from patients with benign ovarian conditions was added to the cell cultures. Cells were incubated for 18h overnight at 37°C in a 5% CO_2_ atmosphere. Supernatants were harvested and analyzed for cytokine levels. In addition, cells were harvested for analysis of surface marker expression by flow cytometry. In order to assess the viability of DC, samples were stained with LIVE/DEAD Fixable Dead Cell Stain Kit from Invitrogen prior to flow cytometric analysis. Neither R848, nor ascites negatively impacted on the cell viability of the DC cultures (S6 Fig).

In order to identify the factors that suppress DC activation, cells were cultured as described above in the presence of neutralizing antibodies against the following factors: IL-10 (mouse IgG2b clone 25209), TGF-β (mouse IgG1 clone 27235), VEGFα (mouse IgG2b clone 26503), LIF (mouse IgG2b clone 9824), IL-6 (mouse IgG2b clone 1936; all R&D) and PGE_2_ (mouse IgG1 clone 2B5; Cayman Europe, Tallinn, Estonia). The antibodies were added to cell cultures either separately or in combinations with a final concentration of 5μg/ml per antibody. Mouse IgG2b clone 20116 (IL-10, VEGFα, LIF and IL-6 neutralizing antibodies) and mouse IgG1 clone 11711 (TGFβ and PGE_2_) isotype control antibodies were also used at 5μg/ml.

### Depletion of IL-10 and PGE_2_ from ascites

Neutralizing antibody against IL-10 (R&D) or PGE_2_ (Cayman Europe) or relevant mouse IgG2b and mouse IgG1 isotype control antibodies were covalently linked to PureProteome N-Hydroxysuccinimide (NHS) FlexiBind Magnetic Beads (Millipore) following the manufacturer’s protocol. Antibody-coupled beads were added to ascites (20μl antibody-conjugated bead slurry per 1 ml ascites) and the samples were incubated overnight at 4°C with continues mixing. After overnight incubation, beads were removed from ascites samples using the PureProteome Magnetic Stand. Ascites depleted of IL-10, PGE_2_ and mock-depleted samples were stored at -80°C until their use in activation assays. The successful depletion of ascites samples was confirmed by sandwich ELISA for IL-10 and PGE_2_, respectively [23].

### Allogeneic Mixed Leukocyte Reaction (MLR)

Naïve CD4+ T-cells were isolated from PBMCs by negative selection using the EasySep^®^ Human Naïve CD4+ T-cell Enrichment Kit (StemCell Technologies) following the manufacturer’s instructions. Enriched naïve CD4^+^ T-cells were labeled with carboxyfluorescein diacetate succinimidyl ester (CFSE) (Molecular Probes, Paisley, UK). For this, cells were resuspended in 1μM CFSE solution at a density of 1x10^7^ cells/ml and incubated at room temperature for 10 minutes. To quench the staining, an equivalent volume of FCS was added for 2 minutes, before cells were washed and seeded into round bottom 96-well plates at a density of 5x10^4^ cells/well.

Monocyte-derived DC were pre-cultured overnight under varying conditions with or without TLR agonists and/or ascites as described above. DC were washed thoroughly before being added to the CFSE-labeled CD4^+^ T cells at ratios of 1:100 or 1:200 (DC: T cells). Samples were performed in triplicates. The MLR cultures were incubated at 37°C in a 5% CO_2_ atmosphere for 6 days. After harvest, cells were analyzed by flow cytometry to assess T cell proliferation by CFSE dilution and T cell polarization by intracellular cytokine staining.

### Flow cytometry

Cells were stained according to the manufacturer’s recommendations with the following antibodies alone or in varying combinations, depending on the experiment: CD14-FITC, CD4-APC and CD45RA-PE (all Immunotools, Friesoythe, Germany), CD40-PE (AbD Serotec, Kidlington, UK), CD86-V450, HLA-DR-PerCPCy5.5, CD1a-PE and CD3-APC/Cy7 (all BD). For surface marker staining, PBS containing 1% FCS and 5mM EDTA (Sigma Aldrich) was used as staining buffer. For intracellular cytokine staining with IFNγ-PerCPCy5.5 (eBioscience), IL17A-PE and IL10-Brilliant Violet 421 (both BioLegend), monensin solution (BioLegend) was added to cell cultures for 6 hours before harvesting. Once harvested, cells were fixed and permeabilized using the fixation and permeabilization buffer set from BioLegend. Staining with FoxP3-Alexa Fluor 647 (BD) was performed with FoxP3 staining buffer set (eBioscience). The samples were analyzed by flow cytometry on a BD FACS Canto II.

### Cytokine analysis

Flow Cytomix^®^ (eBioscience, Hatfield, UK) simplex kits for IL-6 and TNFα were used for the measurement of cytokine levels in cell culture supernatants. The samples were acquired on a BD FACS Canto II. Alternatively, cytokines in cell culture supernatants were measured by IL-6 ELISA (BD) and TNFα ELISA (BioLegend, Cambridge, UK). Absolute cytokine values measured by flow cytomix versus ELISA differed considerably, especially for TNFα. In all experiments, IL-12p40 analysis in cell culture supernatants was performed by ELISA (BD).

PGE_2_ Parameter Assay kit (Arbor Assays, Ann Arbor, MI, U.S.A.), CTLA-4 Platinum ELISA kit (eBioscience), Arginase-1 ELISA kit (Hycult Biotech, Uden, The Netherlands), LIF ELISA kit and VEGFα, CCL18, IL-6, IL-10, TNFα, IFNγ and IL-17 matched antibody pairs (all R&D) were used following respective manufacturers’ protocols for cytokine analysis in peritoneal fluid.

### Statistics

Statistical analysis of activation assay samples was performed using one-way ANOVA Friedman test with Dunn post-test (Prism Program; Graphpad software, La Jolla, USA). In the graphs p values are indicated as follows: * p<0.05; ** p<0.01; *** p<0.0001; n.s. not significant (p>0.05). For statistical analysis of experiments with only two groups, the Wilcoxon signed ranks test was used for paired samples whereas the Mann-Whitney U test was used for unpaired samples. To determine correlations between cytokine levels in ascites and suppression, the distribution of variables was assessed by D’Agostino and Pearson omnibus normality test. Where cytokine levels in ascites samples were distributed normally, correlation to suppression was assessed by calculating the Pearson coefficient using Prism Software. For cytokine levels lacking normal distribution across ascites samples, the Spearman coefficient was calculated instead.

## Supporting information

S1 Fig CD86 up-regulation and cytokine induction in response to TLR stimulation of monocyte-derived DC. Monocyte-derived DC were cultured overnight in complete medium only, or stimulated with 3μg/ml R848, 1μg/ml LPS or 100μg/ml polyI:C. The mean fluorescence intensity (MFI) of CD86 was assessed by flow cytometry. Cytokines were measured in culture supernatants by flow cytomix analysis (IL-6, TNFα) or sandwich ELISA (IL-12p40). n = 12 (12 independent experiments with DC from six different healthy volunteers cultured with ascites from 4 (n = 1), 3 (n = 1), 2 (n = 1) or 1 (n = 3) OC patient); One-way ANOVA (Friedman test with Dunn post test): * = p<0.05; ** = p<0.01; *** = p<0.001; ns = not significant.
(TIFF)

S2 Fig CD86 up-regulation and IL1β induction in response to TLR stimulation is not altered in the presence of OC-associated ascites. Monocyte-derived DC were (A) cultured with medium or (B) stimulated overnight with 3μg/ml R848, 1μg/ml LPS or 100μg/ml polyI:C in the presence of 0%, 10% or 25% of ascites from patients suffering from malignant OC. The following day, the MFI of surface marker CD86 was assessed by flow cytometry and cytokines were measured in culture supernatants by flow cytomix analysis or sandwich ELISA (IL-12p40). 12 independent experiments were performed (n = 12) with DC from six different healthy volunteers cultured with ascites from 4 (n = 1), 3 (n = 1), 2 (n = 1) or 1 (n = 3) OC patient). One-way ANOVA was used for statistical analysis (Friedman test with Dunn post test); * = p<0.05; ** = p<0.01; *** = p<0.001; ns = not significant.
(TIFF)

S3 Fig Correlation of DC activation markers with OC-associated immunosuppressive factors. Levels of proteins in ascites samples are correlated to the suppression of TLR-mediated up-regulation of CD86 and production of IL-6 and IL-12p40. Suppression is expressed in per cent reduction of surface marker and cytokine levels when 10% ascites was added to the cell culture as compared to no ascites present. (A) Correlation between VEGFα levels and CD86 suppression: Pearson r = -0.5524 p = 0.0034 (B) Correlation between Arg-1 levels and CD86 suppression: Pearson r = -0.5513 p = 0.0035; correlation between Arg-1 levels and IL-6 suppression: Pearson r = -0.4527 p = 0.0202 and (C) correlation between TNFα levels and CD86 suppression: Spearman r = 0.5845 p = 0.0017; correlation between TNFα levels and IL-6 suppression: Spearman r = 0.4775 p = 0.0136; correlation between TNFα levels and IL-12p40 suppression: Spearman r = 0.4470 p = 0.0221.
(TIFF)

S4 Fig IL-10 levels in peritoneal fluid from patient with benign ovarian conditions. Protein levels of IL10 in peritoneal fluid collected from patients with benign conditions were measured by sandwich ELISA (n = 3).
(TIFF)

S5 Fig Neutralisation of autocrine PGE_2_ does not alter the induction of TNFα in response to R848. Monocyted-derived DC were stimulated overnight with 3μg/ml R848 with or without PGE_2_-specific neutralizing antibody (5μg/ml). TNFα levels were measured in culture supernatants by sandwich ELISA. n = 3. Wilcoxon matched pairs test; ns = not significant.
(TIFF)

S6 Fig Neither R848, nor ascites negatively impacts the viability of DC. Monocyte-derived DC were cultured overnight in complete medium only (control) or in the presence or absence of 3μg/ml R848 or 25% ascites from patients suffering from malignant OC. The next day cells were harvested and stained with LIVE/DEAD fixable dead cell stain kit and the percentage of live versus dead cells was assessed by flow cytometry. Cells were gated on forward / sideward scatter plots as shown in A and staining with the fixable green-fluorescence dye was analysed as shown in B and C. As control cells were incubated overnight in the absence of fetal calf serum (w/o FCS), which increased the percentage of dead cells from 4.2% (control) to 21.3% (w/o FCS). The data are representative of three independent experiments.
(TIFF)

S1 Table Correlation of CD86 expression and cytokine production by DC with the expression levels of different immunomodulatory factors in OC acites. Correlation of cytokine levels in OC-associated ascites with the suppressive activity of individual ascites samples as assessed by suppression of TLR-mediated CD86 up-regulation or production of the cytokines IL-6, IL-12p40 or TNFα. Distribution of cytokine levels in ascites samples was assessed by d’Agostino and Pearson omnibus normality test. For normally distributed cytokine levels (Arg, VEGFα, IL-6), the Pearson correlation coefficient was calculated. For cytokines lacking normal distribution of levels between ascites samples (LIF, PGE_2_, CCL18, TNFα, IFNγ), the Spearman correlation coefficient was calculated. r = Pearson r or Spearman r coefficient; two-tailed; * = p<0.05 ** = p<0.01.
(TIFF)

S1 Dataset Minimal data sets. The separate sheets contain the minimal data sets for the results shown in the manuscript.
(XLS)
