# Supplementary figures and images for: Interleukin-10 and prostaglandin E2 have complementary but distinct suppressive effects on Toll-like receptor-mediated dendritic cell activation in ovarian carcinoma
Source: PLoS One. 2017 Apr 14;12(4):e0175712. doi: 10.1371/journal.pone.0175712 (PMC5391951; doi:10.1371/journal.pone.0175712)

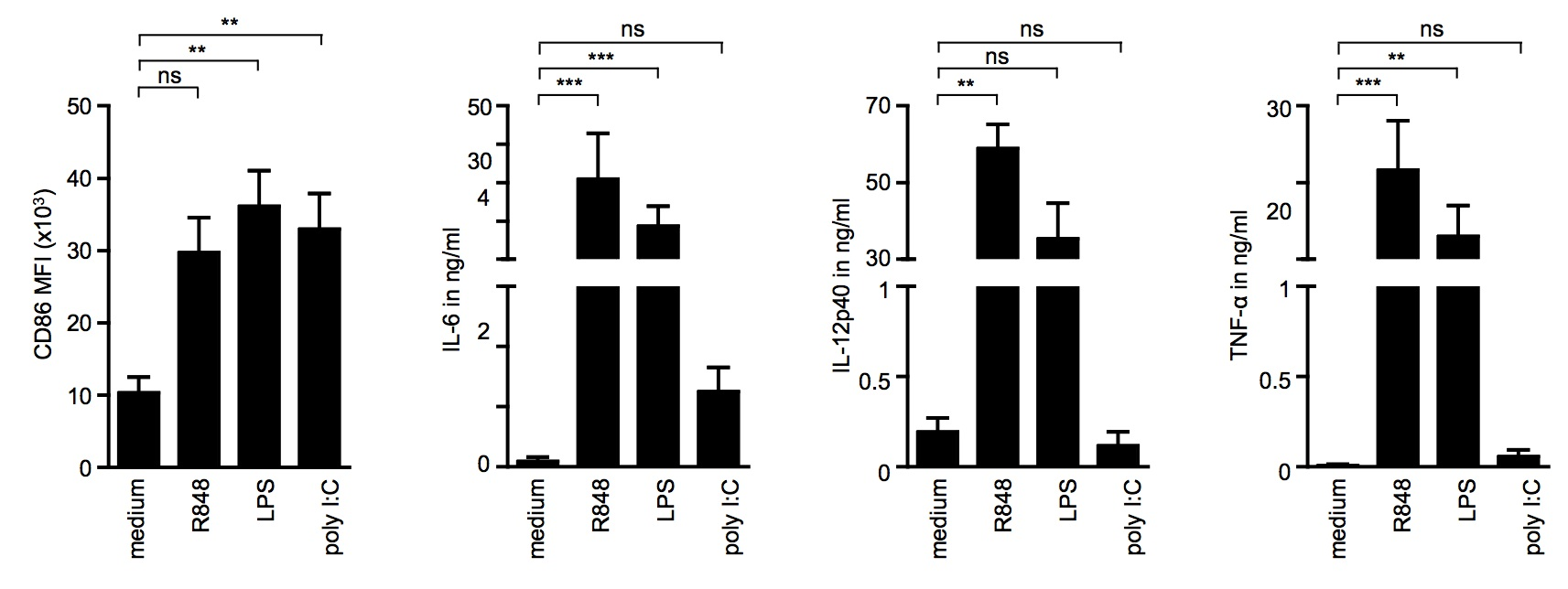

Supplement: S1 Fig — Monocyte-derived DC were cultured overnight in complete medium only, or stimulated with 3μg/ml R848, 1μg/ml LPS or 100μg/ml polyI:C. The mean fluorescence intensity (MFI) of CD86 was assessed by flow cytometry. Cytokines were measured in culture supernatants by flow cytomix analysis (IL-6, TNFα) or sandwich ELISA (IL-12p40). n = 12 (12 independent experiments with DC from six different healthy volunteers cultured with ascites from 4 (n = 1), 3 (n = 1), 2 (n = 1) or 1 (n = 3) OC patient); One-way ANOVA (Friedman test with Dunn post test): * = p<0.05; ** = p<0.01; *** = p<0.001; ns = not significant. (TIFF) [file pone.0175712.s001.tiff]

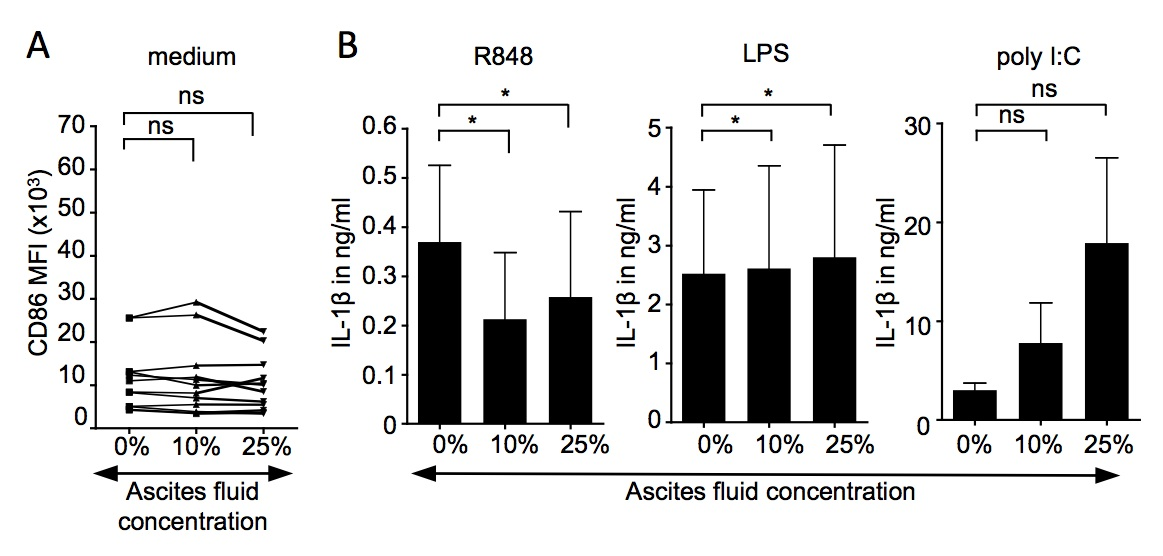

Supplement: S2 Fig — Monocyte-derived DC were (A) cultured with medium or (B) stimulated overnight with 3μg/ml R848, 1μg/ml LPS or 100μg/ml polyI:C in the presence of 0%, 10% or 25% of ascites from patients suffering from malignant OC. The following day, the MFI of surface marker CD86 was assessed by flow cytometry and cytokines were measured in culture supernatants by flow cytomix analysis or sandwich ELISA (IL-12p40). 12 independent experiments were performed (n = 12) with DC from six different healthy volunteers cultured with ascites from 4 (n = 1), 3 (n = 1), 2 (n = 1) or 1 (n = 3) OC patient). One-way ANOVA was used for statistical analysis (Friedman test with Dunn post test); * = p<0.05; ** = p<0.01; *** = p<0.001; ns = not significant. (TIFF) [file pone.0175712.s002.tiff]

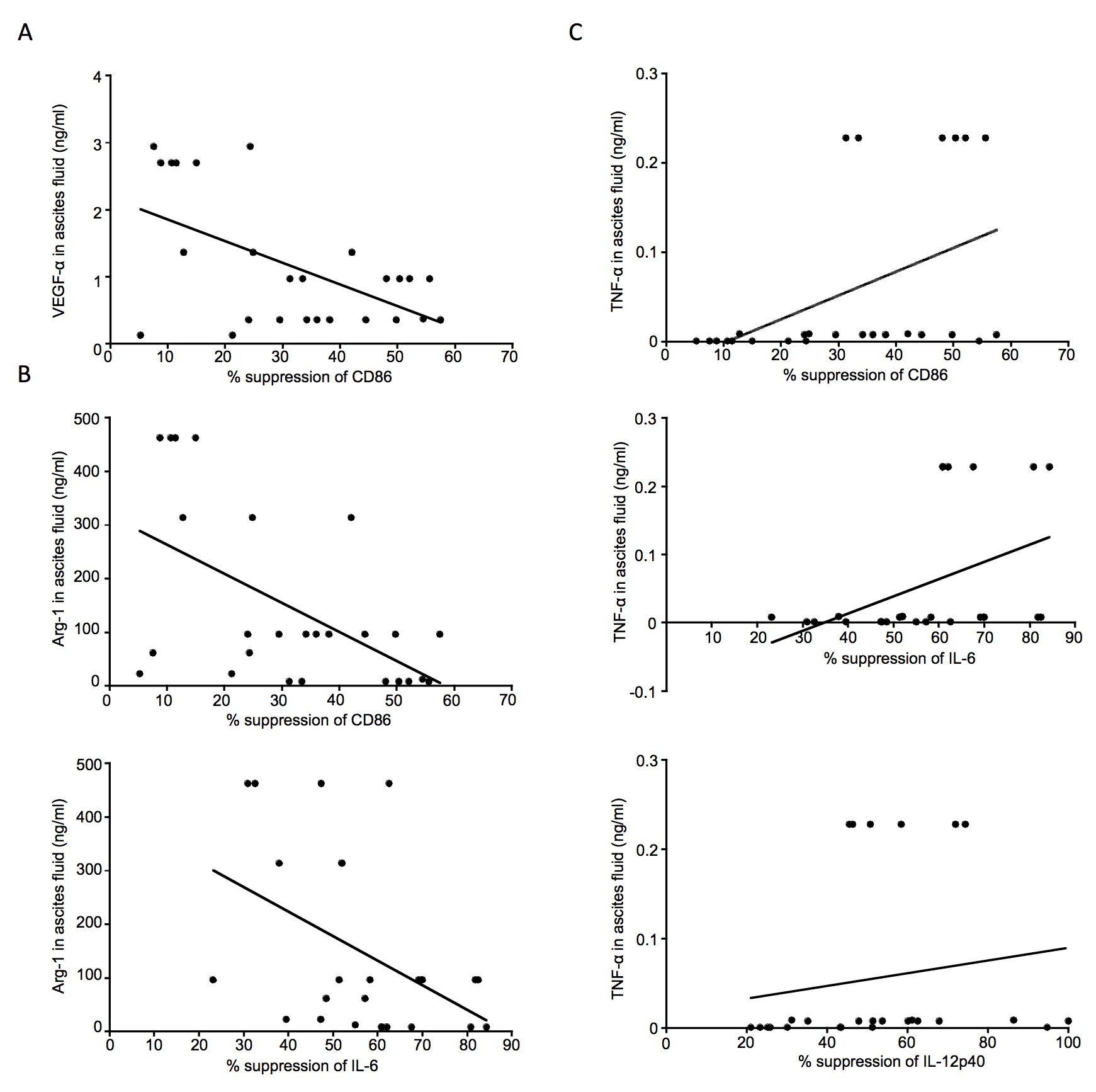

Supplement: S3 Fig — Levels of proteins in ascites samples are correlated to the suppression of TLR-mediated up-regulation of CD86 and production of IL-6 and IL-12p40. Suppression is expressed in per cent reduction of surface marker and cytokine levels when 10% ascites was added to the cell culture as compared to no ascites present. (A) Correlation between VEGFα levels and CD86 suppression: Pearson r = -0.5524 p = 0.0034 (B) Correlation between Arg-1 levels and CD86 suppression: Pearson r = -0.5513 p = 0.0035; correlation between Arg-1 levels and IL-6 suppression: Pearson r = -0.4527 p = 0.0202 and (C) correlation between TNFα levels and CD86 suppression: Spearman r = 0.5845 p = 0.0017; correlation between TNFα levels and IL-6 suppression: Spearman r = 0.4775 p = 0.0136; correlation between TNFα levels and IL-12p40 suppression: Spearman r = 0.4470 p = 0.0221. (TIFF) [file pone.0175712.s003.tiff]

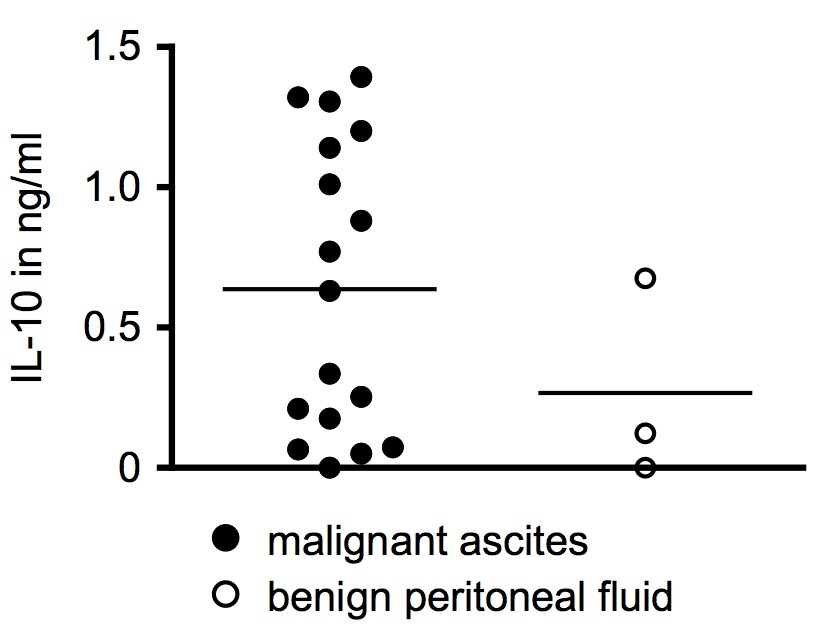

Supplement: S4 Fig — Protein levels of IL10 in peritoneal fluid collected from patients with benign conditions were measured by sandwich ELISA (n = 3). (TIFF) [file pone.0175712.s004.tiff]

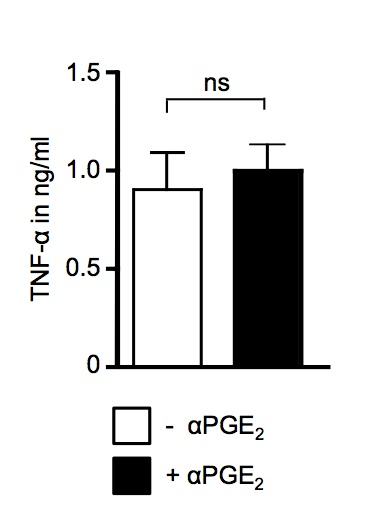

Supplement: S5 Fig — Monocyted-derived DC were stimulated overnight with 3μg/ml R848 with or without PGE2-specific neutralizing antibody (5μg/ml). TNFα levels were measured in culture supernatants by sandwich ELISA. n = 3. Wilcoxon matched pairs test; ns = not significant. (TIFF) [file pone.0175712.s005.tiff]

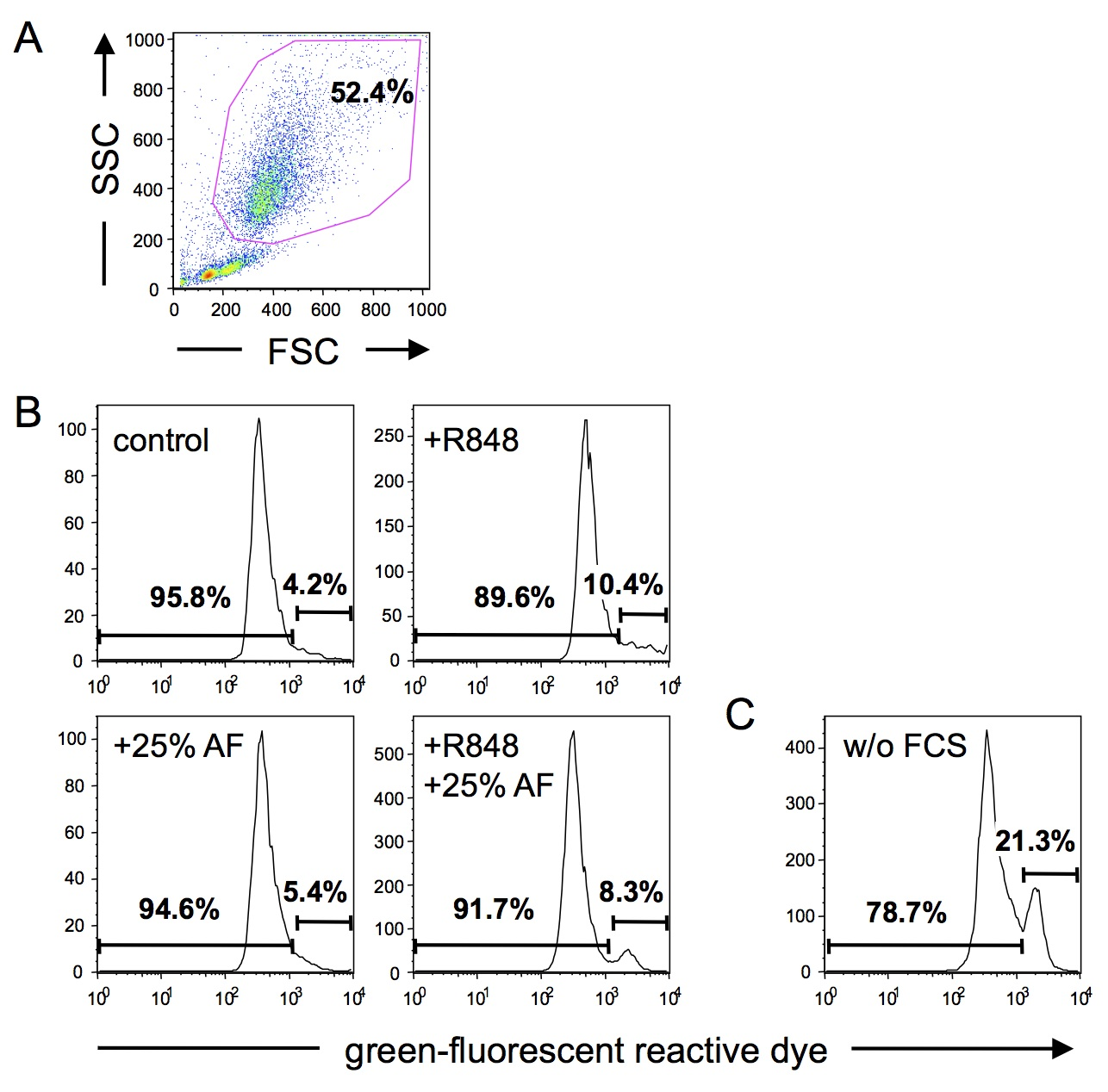

Supplement: S6 Fig — Monocyte-derived DC were cultured overnight in complete medium only (control) or in the presence or absence of 3μg/ml R848 or 25% ascites from patients suffering from malignant OC. The next day cells were harvested and stained with LIVE/DEAD fixable dead cell stain kit and the percentage of live versus dead cells was assessed by flow cytometry. Cells were gated on forward / sideward scatter plots as shown in A and staining with the fixable green-fluorescence dye was analysed as shown in B and C. As control cells were incubated overnight in the absence of fetal calf serum (w/o FCS), which increased the percentage of dead cells from 4.2% (control) to 21.3% (w/o FCS). The data are representative of three independent experiments. (TIFF) [file pone.0175712.s006.tiff]

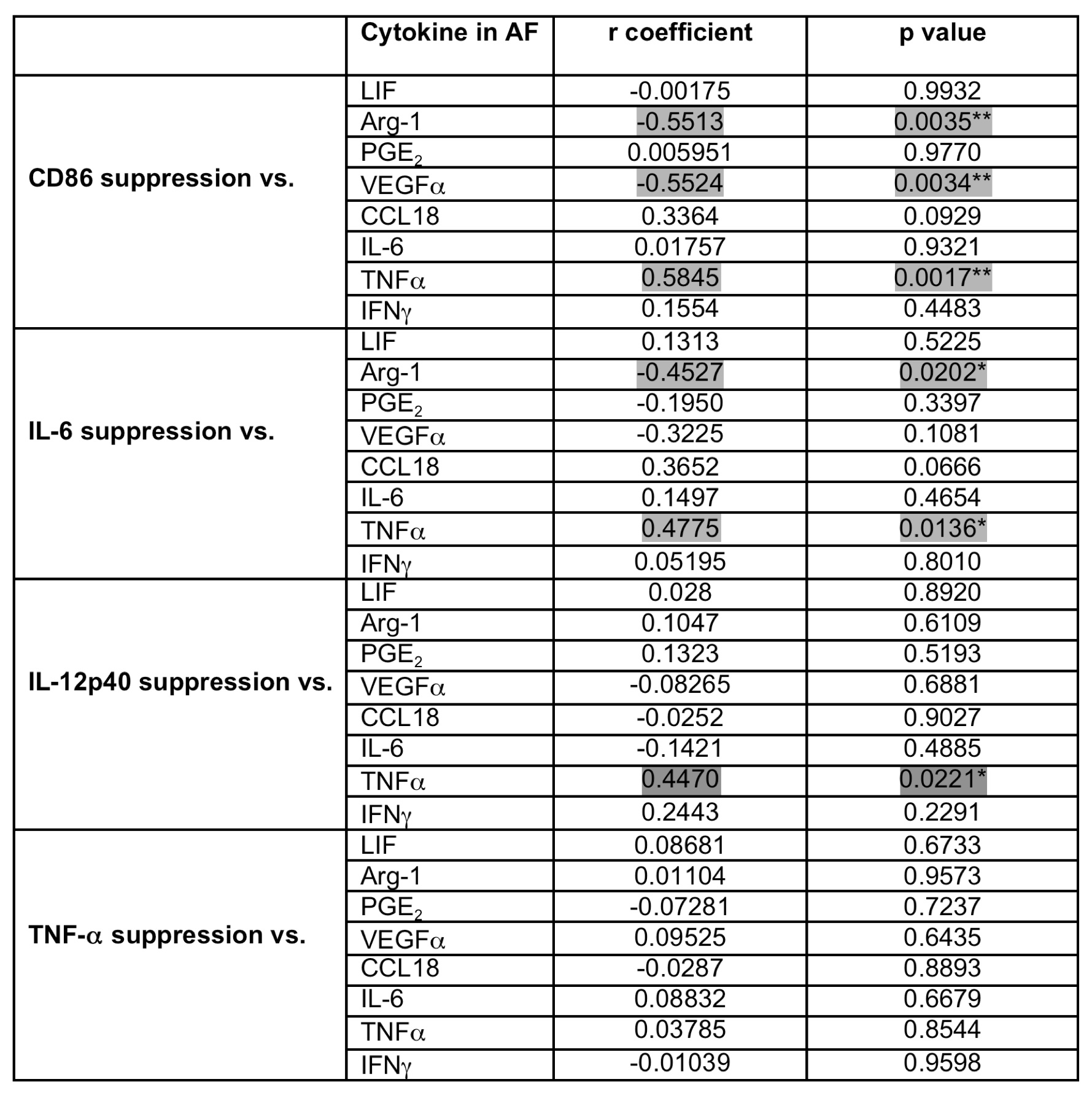

Supplement: S1 Table — Correlation of cytokine levels in OC-associated ascites with the suppressive activity of individual ascites samples as assessed by suppression of TLR-mediated CD86 up-regulation or production of the cytokines IL-6, IL-12p40 or TNFα. Distribution of cytokine levels in ascites samples was assessed by d’Agostino and Pearson omnibus normality test. For normally distributed cytokine levels (Arg, VEGFα, IL-6), the Pearson correlation coefficient was calculated. For cytokines lacking normal distribution of levels between ascites samples (LIF, PGE2, CCL18, TNFα, IFNγ), the Spearman correlation coefficient was calculated. r = Pearson r or Spearman r coefficient; two-tailed; * = p<0.05 ** = p<0.01. (TIFF) [file pone.0175712.s007.tiff]
